# The Saliva a Protection Afforded by Nature against Caries

**Published:** 1901-10

**Authors:** William H. Potter


### Reviews of Dental Literature.

The Saliva a Protection afforded by Nature against Caries. By Dr. A. Michel.[1]

An accurate translation of the entire article is necessary to give an adequate idea of the subject as treated by the author. I shall only be able to give the main statements and some of the conclusions drawn from them. An important statement made by the author is that the cleaning and washing of the teeth to free them from the food remains, and the making slippery the bolus of food constitute the principle functions of the saliva. Its ability to change starch into sugar is only useful in that it facilitates the cleansing process by changing insoluble starch into soluble sugar. Apart from its function as a mechanical cleanser, two other activities are to be mentioned which help preserve the teeth. In the first place, its ability to neutralize acids; in the second place, its antifermentative action. The author gives several reasons in support of his view that the starch-sugar change is only an incidental action of the saliva. He cites the fact that the whale and the dolphin, which wash out their mouths and teeth freely with sea-water, have very poorly developed saliva glands. Also that the carnivora, which do not eat starchy food, have well-developed salivary glands, which cannot be intended for their ability to change starch into sugar. But the most weighty argument is that lower in the alimentary tract such abundant provision is made for the conversion of starch that it seems probable that the work was intended to be done there rather than in the mouth. The author gives an elaborate analysis of normal saliva, and states various changes in health and disease, and then follows an interesting analysis of the drinking-water of the two towns of Würzburg and Lohr; also an analysis of the saliva taken from inhabitants of the two towns.

The results are as follows: The drinking water of Würzburg has fifty times as much calcium oxide as that of Lohr. And the calcium oxide of the saliva taken from inhabitants of Lohr is only

[1] Der Speichel als natürlicher Schutz gegen Caries von A. Michel. Deutsche Monatsschrift für Zahnheilkunde, 4. Juni, 1901.

one-sixth of that found in the saliva of inhabitants of Würzburg. The author is led to the belief that in regions where there is a lack of lime in the drinking-water caries is increased. This comes about in two ways: first, because not enough lime is present for the proper forming of the teeth, and hence they are structurally defective; secondly, the alkalinity of the saliva is lessened and it is not able to properly protect the teeth against the acids formed in the mouth.

After making many analyses of saliva, and noting the degree of alkalinity and also the frequency of caries, the author comes to the following conclusion: where a lessening of caries is found there is found an increase in the amount of sulphocyanic acid and an increase in the alkalinity of the saliva.

William H. Potter.

